# Supplementary material for: A Th2 Cytokine Profile in Appendicular Lavage Fluid Suggests Allergy as a Possible Etiology for Acute Appendicitis
Source: Mediators Inflamm. 2019 Oct 28;2019:8146257. doi: 10.1155/2019/8146257 (PMC6854935; doi:10.1155/2019/8146257)

**Supplementary Figure 1**. Box plots of IL-6 levels according to the different histological categories.


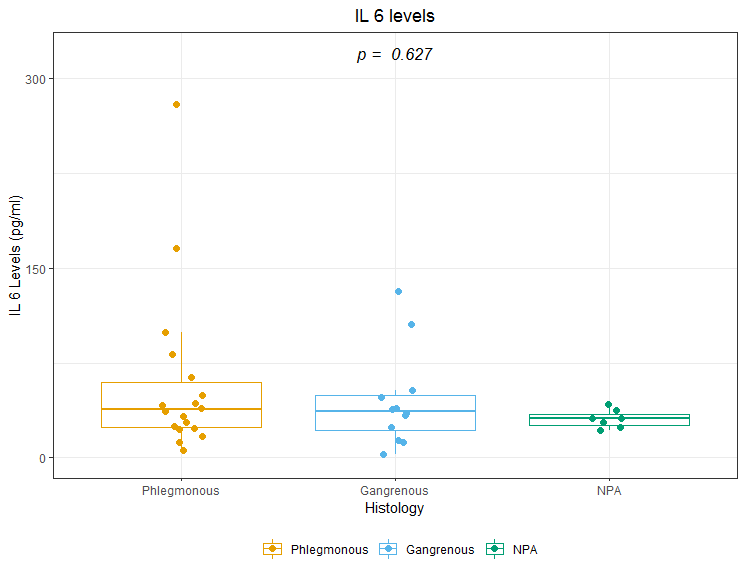

Supplement: Supplementary Materials — A single figure that displays the IL-6 levels in AA samples according to their histological classification. [file 8146257.f1.docx]
